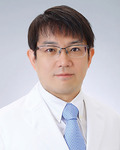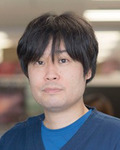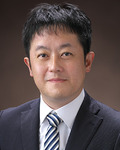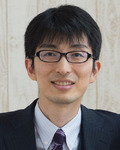# Best Reviewers Award for 2022

**DOI:** 10.1002/deo2.235

**Published:** 2023-04-28

The DEN Open Best Reviewers Award is an annual prize which recognizes the very best reviewers for their high‐quality reviews and dedication. Over 194 scholars served as reviewers in 2022, and we are pleased to announce 20 winners who have been selected based on the following criteria:

1. Invitation acceptance rate: 80% and over.
2. Number of completed reviews: 1.5 or above; How to calculate the number of the articles reviewed:
  - Reviews/Original Articles ——— x1
  - Case Reports ——— x0.5
3. Top 20 reviewers whose average scores for review: eight points maximum and two points minimum, Quality (5‐point scale) + Timeliness (3‐point scale).

Review period: 1 January 2022 to 31 December 2022

**Table:** 

**Akira Dobashi** Department of Endoscopy, The Jikei University School of Medicine, Tokyo, Japan	**Osamu Dohi** Molecular Gastroenterology and Hepatology, Graduate School of Medical Science, Kyoto Prefectural University of Medicine, Kyoto, Japan	**Hiroyuki Endo** Department of Gastroenterology, Japan Community Health Care Organization Sendai Hospital, Miyagi, Japan	**Mitsuru Esaki** Department of Medicine and Bioregulatory Science, Graduate School of Medical Sciences, Kyushu University, Fukuoka, Japan
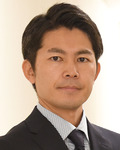	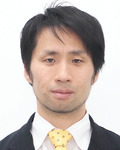	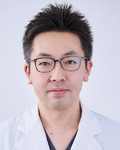	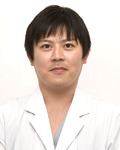
**Natalie Halvorsen** Clinical Effectiveness Research Group, University of Oslo and Oslo University Hospital, Oslo, Norway	**Yusuke Horiuchi** Deparment of Gastroenterology, Cancer Institute Hospital of Japanese Foundation for Cancer Research, Tokyo, Japan	**Ryoji Ichijima** Division of Gastroenterology and Hepatology, Department of Medicine, Nihon University School of Medicine, Tokyo, Japan	**Yusuke Ishida** Department of Gastroenterology and Medicine, Fukuoka University Faculty of Medicine, Fukuoka, Japan
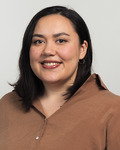	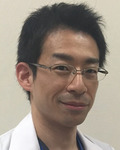	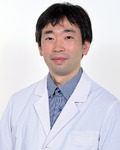	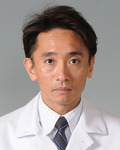

**Table:** 

**Takashi Kanesaka** Department of Gastrointestinal Oncology, Osaka International Cancer Institute, Osaka, Japan	**Tomoyuki Koike** Division of Gastroenterology, Tohoku University Graduate School of Medicine, Miyagi, Japan	**Masaki Kuwatani** Department of Gastroenterology and Hepatology, Hokkaido University Hospital, Hokkaido, Japan	**Mai Makiguchi** Endoscopy Division, National Cancer Center Hospital, Tokyo, Japan
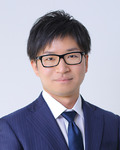	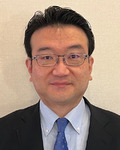	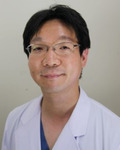	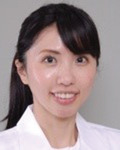
**Yasuaki Nagami** Department of Gastroenterology, Osaka Metropolitan University Graduate School of Medicine, Osaka, Japan	**Teppei Omori** Institute of Gastroenterology, Tokyo Women's Medical University, Tokyo, Japan	**Yuki Tanisaka** Department of Gastroenterology, Saitama Medical University International Medical Center, Saitama, Japan	**Takeshi Tomoda** Department of Gastroenterology, Okayama City Hospital, Okayama, Japan
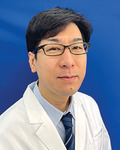	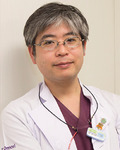	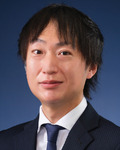	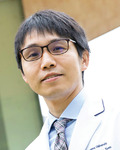
**Ryosuke Tonozuka** Department of Gastroenterology and Hepatology, Tokyo Medical University, Tokyo, Japan	**Yosuke Toya** Division of Gastroenterology and Hepatology, Department of Internal Medicine, Iwate Medical University, Iwate, Japan	**Daisuke Yamaguchi** Department of Gastroenterology, National Hospital Organization Ureshino Medical Center, Saga, Japan	**Kei Yane** Department of Gastroenterology, Tonan Hospital, Hokkaido, Japan